# Mantle Branch-Specific RNA Sequences of Moon Scallop *Amusium pleuronectes* to Identify Shell Color-Associated Genes

**DOI:** 10.1371/journal.pone.0141390

**Published:** 2015-10-23

**Authors:** Rong-lian Huang, Zhe Zheng, Qing-heng Wang, Xiao-xia Zhao, Yue-wen Deng, Yu Jiao, Xiao-dong Du

**Affiliations:** 1 Fishery College, Guangdong Ocean University, Zhanjiang, China; 2 Laboratory of Marine Pearl Culture, Zhanjiang, China; 3 Environment Protection Monitoring Station, Environmental Protection Agency of Zhanjiang, Zhanjiang, China; Institute of Oceanology, Chinese Academy of Sciences, CHINA

## Abstract

*Amusium pleuronectes* (Linnaeus) that secretes red- and white-colored valves in two branches of mantle tissues is an excellent model for shell color research. High-throughput transcriptome sequencing and profiling were applied in this project to reveal the detailed molecular mechanism of this phenotype differentiation. In this study, 50,796,780 and 54,361,178 clean reads were generated from the left branch (secreting red valve, RS) and right branch (secreting white valve, WS) using the Illumina Hiseq 2000 platform. De novo assembly generated 149,375 and 176,652 unigenes with an average length of 764 bp and 698 bp in RS and WS, respectively. Kyoto encyclopedia of genes and genomes (KEGG) metabolic pathway analysis indicated that the differentially expressed genes were involved in 228 signaling pathways, and 43 genes were significantly enriched (P<0.01). Nineteen of 20 differentially expressed vitellogenin genes showed significantly high expression in RS, which suggested that they probably played a crucial role in organic pigment assembly and transportation of the shell. Moreover, 687 crystal formation-related (or biomineralization-related) genes were detected in *A*. *pleuronectes*, among which 144 genes exhibited significant difference between the two branches. Those genes could be classified into shell matrix framework participants, crystal nucleation and growth-related elements, upstream regulation factors, Ca level regulators, and other classifications. We also identified putative SNP and SSR markers from these samples which provided the markers for genetic diversity analysis, genetic linkage, QTL analysis. These results provide insight into the complexity of shell color differentiation in *A*. *pleuronectes* so as valuable resources for further research.

## Introduction

Variations in shell color display in Mollusca species indicate ecological adaptation to environmental factors, which is an evolved superior quality associated with growth performance. Atkinson (1980) reported that natural selection plays an important role in the color polymorphism of *Littorina rudis* and *Littorina arcana*, [1] mangrove snail (*Littoraria filosa*), sand-dwelling whelk (*Bullia digitalis*), [2] freshwater snail (*Pomacea flagellate*), [3] and *Littorina saxatilis*. [4] Although ambient environments can influence shell color variation, a number of previous studies suggested that this phenotypic trait is a genetic control mechanism during lineage evolution of species. [5,6] Shell color heritance has been recently found to supply the molecular marker that assists breeding for high yield and superior quality, which have economic value. [7–10] In particular, pearl production of pearl oyster can be greatly improved by incorporating pigmentation traits during breeding. [11–13]

Organic pigment coloring is initially believed to be one of the most important factors that determine shell color. Previous studies conducted a series of shell pigment observations, and found the pigment substances are diverse in different species. [14–16] Recently, Carotenoids were found to be an important organic matrix in colored aragonite crystal of pearl shell. [17] However, the mollusks are unable to synthesize these pigments; therefore, mollusks absorb and accumulate these pigments from food. [18] Furthermore, the study on the color secretory system within the abalone mantle tissue indicates that pigmentation is controlled by the coordination of patterning events with signaling networks to synchronize the accumulation and secretion of pigmented material. [19] Interestingly, structure coloring determinations are now gained more attention. By using next generation RNA-seq, Bai identify genes involved in nacre color determination in triangle sail mussel *Hyriopsis cumingii* (Lea), which included the biomineralization-related genes. [20]

High-throughput deep sequencing technologies serve as a foundation for gene structure and function research, [21] as well as examining complex molecular mechanisms of disease, [22] structure formation, [23,24] mining genetic or genomic resources, [25] and evolution [26] in aquatic animals. *Amusium pleuronectes* (Linnaeus) is a well-characterized model, which contains two valves, the upper (left) red and the lower (right) white ones. This model was applied to detect the valves secreted by two mantle tissue branches. [27] After eliminating the external interference, the organism’s internal systems were detected by a highly efficient platform, digital expression profiling analysis, and RNA sequences. These valves were compared with systematically characterize differences in mRNA expression levels between mantle tissues that secrete red and white valves. In addition, genes involved in both organic pigment and structural (biomineralization-related) coloring determination factors for further insight into the mechanism behind these remarkably diverse traits in mollusks were identified. Furthermore, putative SNP and SSR markers were identified from these Unigenes, which may provide the markers for genetic diversity analysis, genetic linkage, QTL analysis. Finally, *A*. *pleuronectes*, which is characterized by a larger body, rapid growth, unique muscularity, and palatable quality, has great commercial value in China, the Philippines, Thailand, and Australia. [28] This transcriptomic resource should serve as a solid foundation for future genetic or genomic studies on *A*. *pleuronectes*.

## Materials and Methods

### Experimental animals

In October 2012, animals were sampled from the coastal area of Bebuwan Bay (108°30′-108°50′E, 20°70′-20°90′N), which belongs to the South China Sea, under the jurisdiction of Guangxi province, China. The local fisheries authority enforces a fishing moratorium between June 1 and August 1 to conserve fisheries resources. After the fishing off season, the fishermen were allowed to resume operation in that area. The sampling process does not involve endangered or protected animals. They were purchased from Dijiao Wharf market in Beihai (109.086576°E, 21.486342°N) near the Beibu Gulf, China. The scallops used were 6.8–7.5 cm in shell length and cultured in the aquarium at Lab of Marine Pearl Culture. Two mantle tissue branches of each individual (a total of 10 individuals) were separately dissected from red (R) and white valve (W).

### RNA extraction

Total RNAs of ten mantle tissues were respectively extracted using Trizol reagent (Invitrogen) according to the manufacturer’s protocol. RNA integrity and quantity were evaluated through lab-on-chip analysis using a 2100 bioanalyzer (Agilent Technologies). In the current study, two samples had RNA integrity number values of 7.9 and 7.8. Then, equal amounts of mRNA (each of 2μg total RNA) from five left branches were pooled for a “red valve-secreting tissue”, as well as five right branches for a“white valve-secreting tissue” sample.

### Library construction

The cDNA library was constructed according to the manufacturer’s instructions (Illumina/Hiseq-2000 RNA-seq). Poly(A)^+^ RNA was purified using Sera-mag Magnetic Oligo (dT) beads from total RNA samples. The collected mRNAs were firstly cleaved into fragments. And the prepared buffers containing dNTPs, RNaseH, and DNA polymerase I were used to reverse transcript these fragments to second-strand cDNA purified by QiaQuick PCR extraction kit (Qiagen). After adapter connection, the cDNA fragments (200±25 bp) were purified and sequenced in BGI-Shenzhen via Illumina/Hiseq-2000 RNA-seq.

### Transcriptome analysis

Transcriptome analysis used de novo assembly bioinformatics analysis. Primary sequencing data (or raw reads) produced by the Illumina/Hiseq-2000 platform were evaluated by the quality control (QC) to determine if a re-sequencing step is required. After QC, raw reads are filtered as clean reads, which will be used in Trinity software [29] to assemble unigenes (i.e., R-unigene and W-unigene). Multiple samples from the same species were sequenced, and then the unigenes were obtained from the assembly of each sample. These unigenes were further processed by sequence splicing and redundancy removal to produce the longest possible non-redundant unigene (All-unigene). Finally, the All-unigene was obtained from each sample assembly. All-unigene sequences were first aligned by Blastx to protein databases, such as Nr, SwissPro, KEGG, and COG, with E-value <10^−5^. In addition, the function of All-unigenes was assigned by their corresponding best-hit function-known proteins from each database. The algorithm of reads per kilobase of unigene per million mapped reads (RPKM) [30] was utilized to calculate the expression levels of all unigenes in each sample. And then the differentially expressed genes and performed gene ontology (GO) and pathway enrichment analyses were identified. P-value corresponds to the results of differential gene expression test. False discovery rate (FDR) is a method that determines the P-value threshold in multiple tests. The threshold (FDR≤0.001 [31] and log_2_ *Ratio*≥1 absolute value) was used to assess the significance of gene expression differences.

### Validation of RNA-Seq analysis by real-time quantitative PCR

Real-time quantitative PCR (RT-qPCR) was designed to verify the differences in gene expression of selected target gene observed during RNA-Seq analysis. The mantle tissues of *A*. *Pleuronectes* (Linneus) for RT-qPCR were collected from 5 individuals. Both sides (red and white) of mantle were separately collected. The cDNA was synthesized using M-MLV First Strand cDNA Synthesis Kit (Invitrogen). Primers designed for each gene were present in S1 Table. The qRT-PCR was performed using DyNAmoColorFlash SYBR Green qPCR Kit (Thermo Scientific, USA) according to the manufacturer’s protocol and done on the 7500 Real-time PCR system (ABI Applied Bisosystems, USA). After amplification, fluorescent data was converted to threshold cycle values (CT). The concentration of the template in the sample was determined by relating the CT value to the standard curve. Target gene transcript levels were normalized against reference gene transcripts levels. GAPDH was used as the reference gene. Statistical analysis Quantitative data was expressed as mean ± S.E.M.

## Results and Discussion

### Sequencing and assembly

Poly(A)^+^-enriched mRNA from RS and WS of *A*. *pleuronectes* were sequenced using Illumina/Hiseq-2000 to obtain 54,361,178 and 50,796,780 clean reads, respectively. Detailed GC content, Q20, and unknown bases are shown in S2A Table. Data were archived at the NCBI Sequence Read Archive (SRA) under accession SRA297257. Assembly of the reads produced Trinity_original and unigenes, which could be clustered into two databases. A total of 159,521 All-unigenes were obtained, which ranged from 200 bp to 23,337 bp in size. Furthermore, All-unigene N50 was 979 bp (S2B Table). Gap distribution was below 5% (S2C Table). These results showed that the obtained unigenes were suitable for further annotation.

### Functional annotation of unigenes

**Fig 1:**
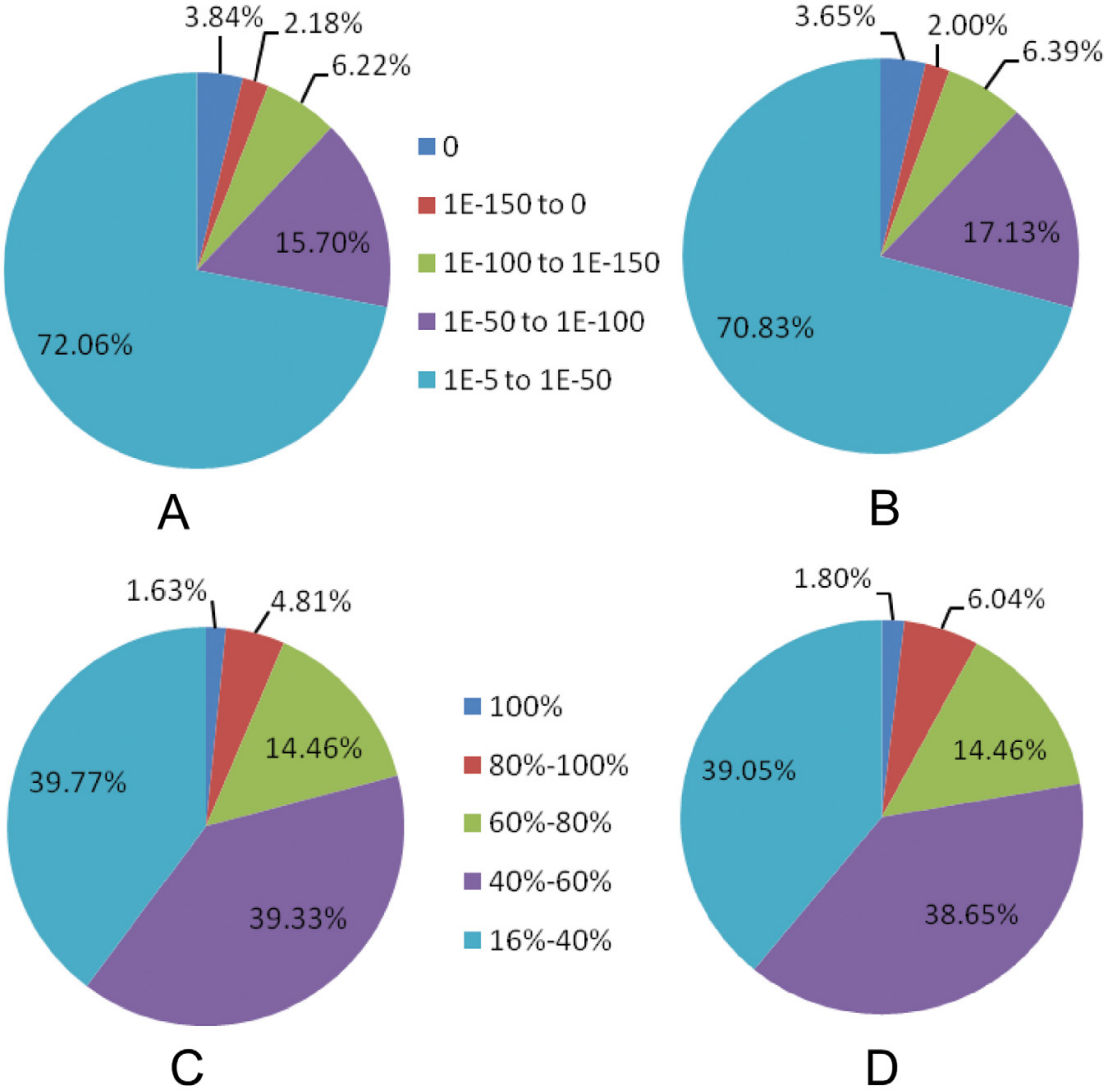
The annotation of assembled All-unigenes. The results of sequence similarity examination conducted against the NCBI Nr (A and B) and Swiss–Prot protein databases (C and D) were shown, respectively. The legend in the middle of A and B represents the E-value and the one between C and D represents the percentage of different identity.

For the annotation of assembled All-unigenes, a sequence similarity examination was conducted against the NCBI Nr and Swiss–Prot protein databases using BLASTx software [32] with a cutoff E-value of 1e-5. A total of 48,597 All-unigenes had significant similarities to known protein databases, of which 46,837 and 41,974 unigenes shared homologous proteins from the Nr and Swiss-Prot databases. Moreover, E-value and similarity distributions of best hits in the Nr and Swiss–Prot databases were analyzed. According to the Nr result, unigenes with significant homology (E-value<1e-50) and high identity (greater than 80%) accounted for 27.94% and 6.43% of all matched ones (Fig 1A and 1C). In the Swiss-Prot result, unigenes with significant homology and high identity accounted for 29.17% and 7.84% of all matches (Fig 1B and 1D).

**Fig 2:**
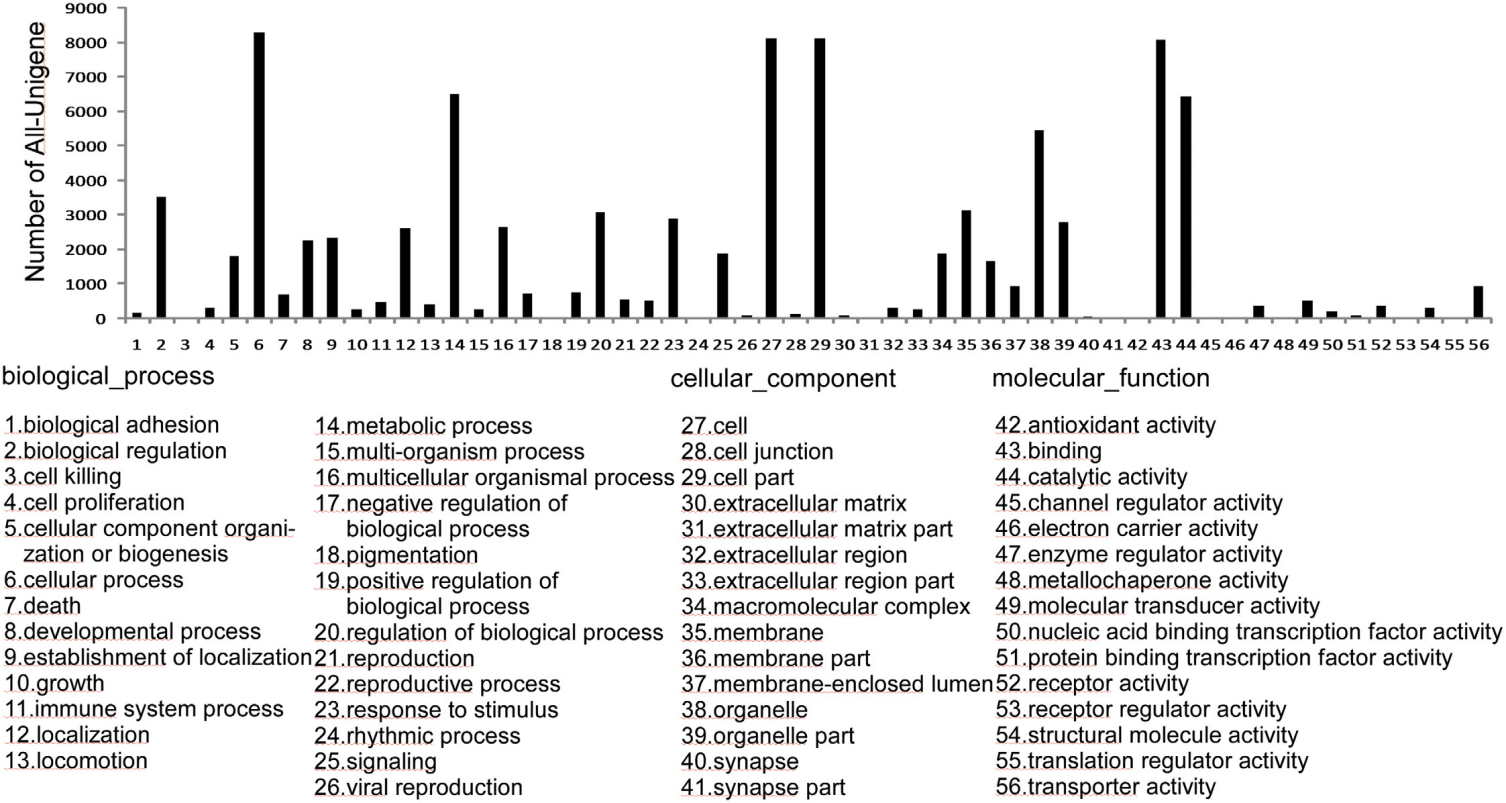
Gene ontology of genes annotated with GO terms.

The GO database is a major bioinformatics innovation that aims to standardize the representation of gene and gene product attributes across species and databases. As shown in the annotation results, 14,738 unigenes were assigned to one or more GO terms. In total, 94,147 GO assignments were obtained: 43,523 were assigned to biological process, 17,524 were assigned to molecular function, and 33,100 were assigned to cellular component (Fig 2). Under the biological process category, cellular process (8,293; 18.93%) was the largest group, which was followed by metabolic process (6,513; 14.96%), biological regulation (3,523; 8.09%), and biological process regulation (3,091; 7.10%). Under the cellular component category, 8,113 (46.30%) unigenes were assigned to cell or cell part, followed by organelle (5,449; 31.09%) and membrane (3,144; 17.94%). For the molecular function category, binding (8,093; 24.29%) was the largest group, which was followed by catalytic activity (6,431; 19.43%), transporter activity (954; 2.88%), and molecular transducer activity (535; 1.62%).

**Fig 3:**
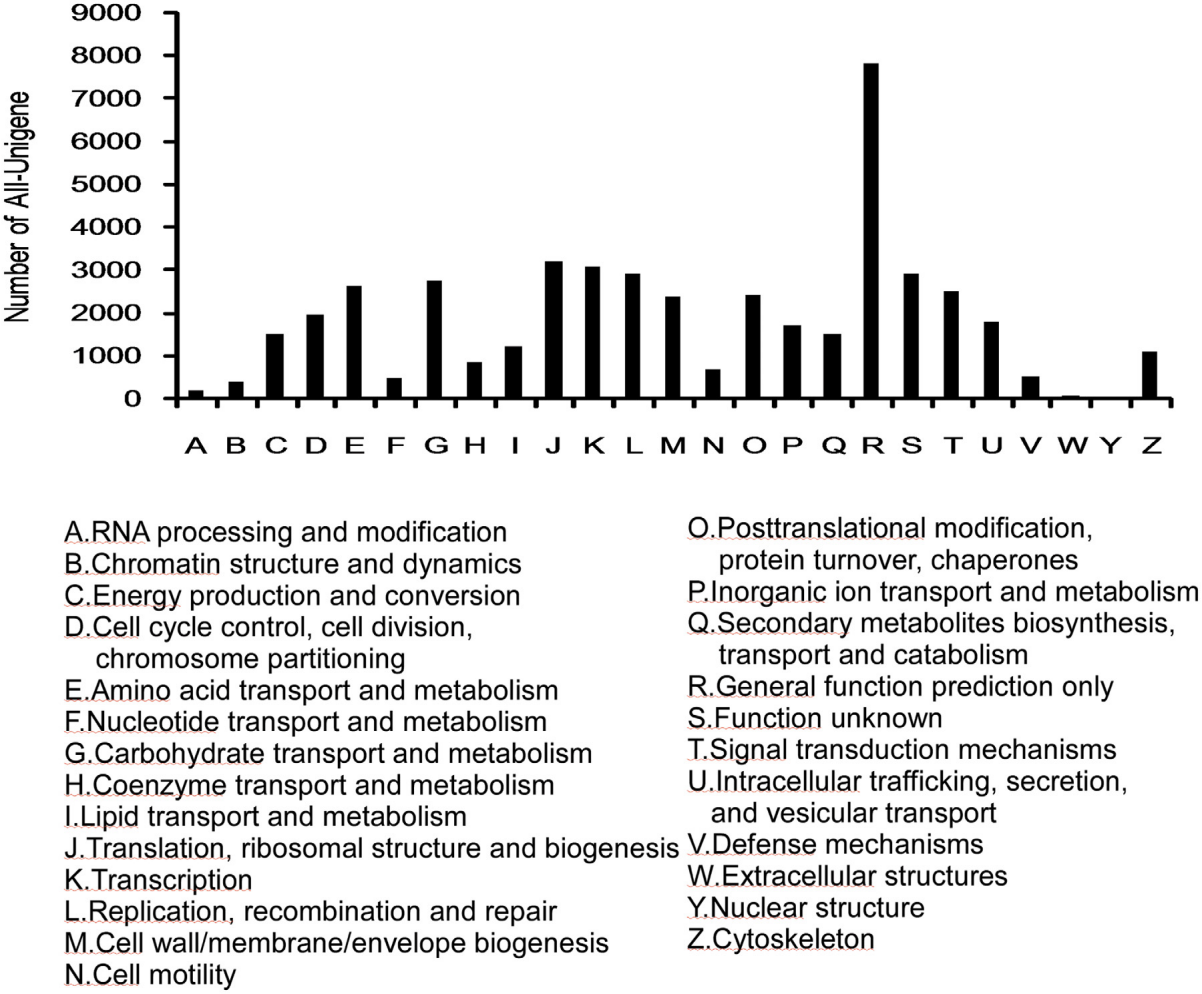
Clusters of orthologous groups annotated with COG terms.

The protein database of clusters of orthologous groups (COGs) aims to systematically classify complete complement of proteins (both predicted and characterized) encoded by complete genomes or transcriptome. In this research, 46,276 All-unigenes were assigned to COG terms. For COG functional classification, general function prediction was predominant (16.90%), followed by translation, ribosomal structure, and biogenesis (6.63%). Extracellular structures (0.09%) and nuclear structures (0.017%) were the least represented COG terms (Fig 3).

### KEGG and GO enrichment analyses of differential expression genes in red (RS) and white (WS) valve-secreting tissues

Left and right mantles secrete red and white valves in *A*. *pleuronectes*. Genes showing differential expression between the two samples were identified using an algorithm developed by Audic et al.. [33] The edgeR software was used to identify the DGEs with critical thresholds (P-value <0.05, fold >2, FDR<0.05). The results suggested that 6,131 genes were significantly highly expressed in RS, while 3,064 genes were lowly expressed, compared with that in WS. In RS, 1,105 specific expressed unigenes were observed, but only 337 of them were observed in WS. These specific expressed unigenes were analyzed based on KEGG cluster analysis. The red shell-specific pathways (93 specific pathways, data not shown) were higher than the white shell-specific pathways [e.g., non-homologous end-joining, cell adhesion molecules (CAMs), fat digestion and absorption, fatty acid metabolism, sulfur metabolism, and MAPK signaling pathway]. In addition, KEGG metabolic pathway analysis indicated that differentially expressed genes were involved in 228 signaling pathways, and 43 genes revealed significant differences (P<0.01). Among these pathways, the ribosome was the most significantly enriched pathway (P-value 3.24e-49), which was followed by oxidative phosphorylation (P-value 2.30e-14).

Among the enriched gene sets of GO in these comparisons, 3,440 of 9,195 genes belonged to GO biological process, 2,007 of 9,195 genes belonged to GO cellular component, and 1,337 of 9,195 genes belonged to GO molecular function in RS-vs-WS. Among these genes, ribonucleoprotein complex, ribosomal subunit, small ribosomal subunit, macromolecular complex, large ribosomal subunit of cellular component, structural molecule activity, cytokine activity of molecular function and gene expression of biological process revealed significant differences (corrected P<0.01) in RS than those in WS (data not shown).

### Identification of genes involved in organic pigmentation

In higher animals, the deposition or aggregation of coloring matter in an organism, tissue, or cell are systematically dissected by a pigment cell called melanocyte, which is an ideal system for genetic, molecular, and cellular analysis. [34–36] A total of 37 identified genes were involved in pigmentation (GO: 0043473), however, only one gene, transcription factor *abd*-*b*, showed significantly higher expression in RS than in WS. The melanogenesis signal pathway contains 53 genes with significant differences in *A*. *pleuronectes*, of which 32 genes exhibited significantly higher expression in RS than in RS. The number of other crucial enzymes was limited during melanin formation in higher animal cells, e.g., tyrosinase-related protein 1 [EC:1.14.18.-] and dopachrome tautomerase [EC:5.3.3.12]. These results suggested the possibility of another available pigmentation mechanism in lower animals. In insect cuticle sclerotization [37] and mollusk shell periostracum, [38,39] the quinone process mainly catalyzed by tyrosinase(TYR) is believed to be a process that embeds pigments into the conchyolin of the shell. TYRs in some mollusk species have been recently reported to be related to crystal shell structures (data shown below). [40]

**Fig 4:**
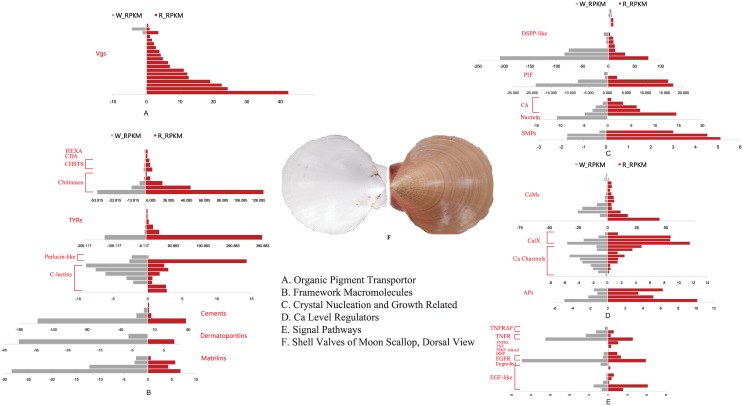
Crystal formation-related genes differently expressed in the RS and WS. The macromorphology of white shell (left) and red shell (right) was shown in F. And the different expressed genes between RS and WS involved in organic pigment transportor (A), framework macromolecules (B), crystal nucleation (C), Ca2+ level regulators (D) and relative signal pathways (E) were represented respectively.

The presence of Carotenoids is related to body colors that appear in tissues of many aquatic animals. [41–43] Mollusk shell contains various pigments, which are commonly carotenoids and porphyrins. However, mollusks are unable to synthesize carotenoids. Therefore, carotenoids are absorbed from food and modified to other forms during accumulation. [18] The carrier, storage, and lipid transfer protein (LLTP) superfamily of pigments mediate the intracellular uptake of lipids, proteins, vitamins, and carotenoids, which include the large cytosolic subunit of the microsomal triglyceride transfer protein (MTP), vertebrate apolipoprotein B (apoB), vitellogenin (Vg), and insect apolipophorin II/I precursor (apoLp-II/I). [44] 47 genes were found, including MTP, Vtg, and apoLp. Notably, we found 33 expressed Vg genes (S3 Table, S1 Fig), of which 20 showed significantly different expression between RS and WS. Moreover, 19 of these genes presented higher expression level in RS (S3 Table, Fig 4A).

### Biomineralization-related genes

There are fewer reports on shell forming-relevant genes in *A*. *pleuronectes*. In this work, the unigenes were compared with the biomineralization-related (BMR) genes from the GenBank database. A total of 687 unigenes were identified, of which 144 were significantly differentially expressed (S3 Table). According to Mann’s shell organic matrix model, two major categories of those shell matrix molecules are defined as framework macromolecules, including insoluble or poorly soluble proteins, polysaccharides or mucopolysaccharide, and soluble acid molecules. These macromolecules regulate the nucleation, orientation, growth, and termination of the CaCO_3_ crystal. [45] However, apart from those well-known organic matrices in shells, upstream signal factors [46] and transporters of minerals [47] should be considered.

Framework macromolecules in shell matrix are considered to play a vital role in biomineralization. The dynamic balance of the chitin framework seems to be pursued by the moon oyster, which is similar to other shelled mollusks and crustaceans. [48] Although 37 chitin synthesis- and degradation-related genes were identified, all differentially expressed genes (nine genes) had higher levels in RS than that in WS (S3 Table, Fig 4B). A similar expression pattern was observed in tyrosinases (TYRs), and seven among 18 patterns were annotated (S3 Table, Fig 4B). Aguilera [40] reported that the TYR gene family has expanded independently in pearl oysters and the Pacific oyster; in addition, their markedly different expression profiles indicate their shell formation function. Zhang also found that TYR may participate in shell matrix maturation by oxidation in the Pacific oyster. [24] Meanwhile, 26 C-lectin genes were annotated (S3 Table). Interestingly, among the seven differentially expressed genes, two genes were similar to *Apis mellifera* (Unigene129630) and *Plutella xylostella* (Unigene124011), which presented higher levels in RS; however, the remaining genes were homologs of mollusk species (S3 Table, Fig 4B). Previous studies indicated that the C-lectin domain is critical for mineralization, but the functional nature of C-lectin and its related genes have not been elucidated. They generally align with other functional domains, which make them as novel genes of mineralization matrix proteins, similar to the echinoderm endoskeleton matrix protein SM50 [49] and abalone shell matrix protein perlucin. [50] Two perlucins were identified: one was similar to *Haliotis diversicolor*, while the other was homologous to *Mytilus galloprovincialis*. These perlucins presented different expression patterns (S3 Table, Fig 4B). In addition to this, nine matrilin genes in *A*.*Pleuronectes* were identified, and five of them had markedly different expression levels (S3 Table, Fig 4B). In higher animals, matrilin supports matrix assembly by connecting fibrillar components and mediating interactions in the cartilage. [51] In the present study, the fibronectin gene family, which is an important shell matrix in some mollusk species, [23,24] was annotated, but apparent differential expression was not detected between the two samples. Moreover, four members of 17 annotated cement genes presented dominant expression levels in WS (S3 Table, Fig 4B). Cement in *A*. *pleuronectes* is serine-rich, which is similar to *Phragmatopoma californica* but in contrast to those found in pearl oyster (i.e., serine, glycine, and glutamine-rich proteins). [23] The expression level of two dermatopontin genes in WS was 5 to 60 times than that in RS (S3 Table, Fig 4B). In some mollusk species, dermatopontin is suggested to function during shell matrix assembly. [52,53] From the aforementioned results, scallop may select mineralization framework matrix proteins to be dominant in its two different valves or at least secrete different ECMs using the two branches of mantle. Chitin and macromolecules were catalyzed by TYRs for the red valve, whereas C-type lectin, matrilin, cement, and dermatopontin were catalyzed for the white valve. This arrangement may possibly contribute to shell coloring; chitin easily adsorbs pigments and has been applied to food engineering applications. [54]

A total of 146 potential genes directly related to crystal nucleation and growth were identified (S3 Table). There were 99 dentin sialophosphoprotein-like genes in *A*.*pleuronectes* (*ApDSPPs*) that have been annotated, and 12 of these genes were differentially expressed (S3 Table, Fig 4C). One *ApDSPP* (Unigene68675) presented the highest expression level (RPKM = 208.541), which contains abundant serine and aspartic acid composition similar to DSPPs in mammals. DSPP (N-linked glycoprotein) and its cleaved products, namely, dentin phosphoprotein (DPP) and dentin sialoprotein (DSP), in mammals are essential for dentin mineralization. Mutation in/or knockout of the DSPP gene results in mineralization defects in dentin and/or bone. [55,56] This large group of DSPP-like genes presented in *A*. *pleuronectes* show that some common features during biomineralization are shared by this mollusk species and higher animals. Another glycoprotein, called PIF, also undergoes proteolytical process, which forms two products, i.e., Pif97 and Pif80; these products have different functions in shell nacre layer formation. [57] All four of 12 PIF annotated genes were remarkably highly expressed in RS (S3 Table, Fig 4C). Meanwhile, eight carbonic anhydrase (CA) genes and one nacrein gene were identified (S3 Table). Among these genes, five CA genes were highly expressed in RS, whereas nacrein presented lower levels in RS (S3 Table, Fig 4C). Nacrein was reported to be a rapid evolutionary product of CA, which specifically supplied the essential element HCO_3_^-^ of crystal nucleation through CO_2_ catalysis in mineralization. [58] Uncoordinated expression of nacrein and other CAs indicated their differential biological functions in *A*.*pleuronectes*. The result of this project showed that BMSP, shell matrix proteins, and perlucin were highly expressed in RS (S3 Table, Fig 4C), which have been reported to directly participate in crystal formation. [50,59,60] In summary, DSPP homologs utilized by moon oyster require further investigation. Those mollusk-specific genes, such as PIF, BMSP, and shell matrix proteins, share a common feature, that is, aspartic acid is believed to be directly involved in crystal nucleation. Their high expression level in RS indicated that the reinforced crystal formation capability should easily stock more macromolecules, such as framework elements and pigments in the shell.

Shells of bivalve animals are calcium metabolism products. The most widely discussed modes of ion uptake for mineralization purposes involve ion transporters and channels located in membranes, a pathway within a cell, and at the mineralization site. A total of 80 Ca^2+^ transport-related genes (CTRs), especially from membrane channels, are found in *A*. *pleuronectes* (S3 Table). Ca exchangers (Calx) in moon scallop were more active in the red valve-secreting branch of mantle. Two of these exchangers were highly similar to that in mammal species (Nr E-value>1E-80) (S3 Table, Fig 4D). Cells expel Ca^2+^ with ATP-driven pumps and Na-Ca exchangers. [61] Interestingly, seven Ca channels homologous to *Lymnaea stagnalis* had higher expression in WS; by contrast, one channel that was highly similar to *Loligo bleekeri* (Nr E-value = 1E-125) presented the highest level in RS (S3 Table, Fig 4D). Despite their differential expression, all had high calcium-buffering capacity in neurons, [62,63] which was distributed in the mantle margins of scallop species. [64] Salmodulin (CaM) is another important constituent of cellular Ca^2+^ regulation, and previous studies showed its close relationship with biomineralization. [65,66] Despite large CaM group members, the mollusk homologous *CaM* found in *A*. *pleuronectes* presented no difference in expression in RS and WS (S3 Table, Fig 4D). Twelve alkaline phosphatases (APs) were identified, of which four showed higher expression level in RS (S3 Table, Fig 4D). All APs in *A*. *pleuronectes* were highly similar to PFAP in pearl oyster, *Pinctada fucata (martensii)*, which may participate in calcium transportation; this result was based from sequence analysis, in situ hybridization, and in vivo studies. [67]

Finally, signal pathways related to mineralization, especially growth factors (GFs) and their receptors, despite the limitations of studies on invertebrate species, were studied in this project. Limited information about these cellular regulators during mineralization has been obtained from sequence analysis, [68] genomic survey, [69] and in vivo and in vitro studies. [70] However, these analogues were hypothesized to have a potential use in the regeneration of living bone for potential clinical use. Notably, 125 genes in the tumor necrosis factor (TNF) signal pathway were detected, including TNF, TNF receptor-associated factor, TNF receptor, and TNF-induced proteins (S3 Table, S2 Fig). The only TNF gene that was highly similar to TNF in human (E-value = 1E119) was detected and significantly expressed in RS (S3 Table, Fig 4E). TNF in higher animals is a potent substance in bone remodeling [46], and several studies suggested that TNF played immunity function in mollusk [71]. The TNF signal pathway may participate in defense systems that trigger inflammatory reaction, which initiates ECM crossing-linking. Furthermore, 54 members of EGF-like substances were identified, one of which was highly similar to *Brugia malayi* (E-value = 1E-148), which showed significantly higher level in RS (FDR = 3.81E-213) (S3 Table, Fig 4E). Only one bone morphogenetic protein (BMP) was identified at high levels in RS. BMP members are reported to play a crucial role in the formation of the shell nacre and prismatic layer; the result was evidenced by expression profiling and RNA interference technology. [72,73]

To validation the authenticity and functions of those genes mentioned above, ten genes were selected randomly for performing the qRT-PCR. The different expression between RS and WS was in union with the RNA-seq data, which demonstrated the accuracy of RNA-seq analysis (S3 Fig).

### The identification of Simple Sequence Repeats (SSRs) and Single Nucleotide Polymorphisms (SNPs)

Misa [74] was applied to screen 159,521 unigene sequences with total size of 106.6Mb, and 15,252 SSRs were detected, which were located in 13,299 unigenes. Among them, 1,632 unigenes contained more than 1 SSRs. Among the distribution of SSR types, the number of Mono-nucleotide is the highest, followed by the Di-nucleotide type, specially, AT/AT type is notable more than others (S4 Fig). 60,167 SNPs and 33,257 Indels were totally identified by using SOAPsnp between RS and WS. These results may provide valuable resource for further analysis, e.g. genetic diversity analysis, genetic linkage and QTL analysis.

## Conclusions

This study investigated the transcritome profile from two branches of mantle tissues in Moon Scallop *A*. *pleuronectes*. The differentially expressed genes identified from RS and WS involve in organic pigmentation and structural (biomineralization-related) coloring determination along with upstream signal factors, which corporately result in the differentiation of two side shells. Therefore, the Moon Scallop could be used as a model for further investigation of the mechanism of shell formation and phenotype differentiation in mollusk.

## Supporting Information

S1 Fig The alignment analysis of vitellogenin. Mye, Mizuhopecten yessoensis (KC138552); Can, Crassostrea angulata (JX218047); Mno, Mimachlamys nobilis (JN638064); Afa, Azumapecten farreri (GQ227743); Pma, Pecten maximus (AM943022); Hdi, Haliotis discus hannai (AB360714); Cgi, Crassostrea gigas (AB084783)
(EPS)

S2 Fig The alignment analysis of tumor necrosis factor receptor-associated factor (TRAF). TRAF6 Esc, Euprymna scolopes (AY956816); TRAF6 Afa, Azumapecten farreri (DQ350773); TRAF3 Pfu, Pinctada fucata (JQ898347); TRAF6 Mye Mizuhopecten yessoensis (JX123315); TRAF6 Mga, Mytilus galloprovincialis (KC994893).
(EPS)

S3 Fig Differential expressioned genes between RS and WS based on RNA-seq and validated by qRT-PCR. A total of 10 genes was successfully validated by the qRT-PCR analysis. No genes showed inconsistent expression pattern with the transctriptome expression results. The Y-axis shows fold change of expression between RS and WS.
(EPS)

S4 Fig The number of different type SNPs identified from all the Unigenes. (EPS)

S1 Table Primer sequence for qRT-PCR. (DOCX)

S2 Table Sequencing and assembling of the unigenes identified in this study. (DOCX)

S3 Table Different expressed genes of shell color-associated genes identified from the WS and RS in *A*. *pleuronectes* (Linnaeus). (XLSX)

## References

[1] Atkinson WD, Warwick T. The role of selection in the colour polymorphism of Littorina rudis Maton and Littorina arcana Hannaford-Ellis (Prosobranchia: Littorinidae). Biological Journal of the Linnean Society. 1983;20: 137–151.

[2] Heller J. Shell colour variation in Bullia digitalis, a sand-dwelling, intertidal whelk (Gastropoda: Prosobranchia). Biological Journal of the Linnean Society. 1992;46: 247–258.

[3] Reed WL, Janzen FJ. Natural selection by avian predators on size and colour of a freshwater snail (Pomacea flagellata). Biological Journal of the Linnean Society. 1999;67: 331–342.

[4] Sokolova IM, Berger VJ. Physiological variation related to shell colour polymorphism in White Sea *Littorina saxatilis* . Journal of Experimental Marine Biology and Ecology. 2000;245: 1–23.

[5] Newkirk GF. Genetics of shell color in *Mytilus edulis* L. and the association of growth rate with shell color. Journal of Experimental Marine Biology and Ecology. 1980;47: 89–94.

[6] Brake J, Evans F, Langdon C. Evidence for genetic control of pigmentation of shell and mantle edge in selected families of Pacific oysters, *Crassostrea gigas* . Aquaculture. 2004;229: 89–98.

[7] Petersen JL, Baerwald MR, Ibarra AM, May B. A first-generation linkage map of the Pacific lion-paw scallop (Nodipecten subnodosus): Initial evidence of QTL for size traits and markers linked to orange shell color. Aquaculture. 2012;350–353: 200–209.

[8] Zou K, Zhang D, Guo H, Zhu C, Li M, Jiang S. A preliminary study for identification of candidate AFLP markers under artificial selection for shell color in pearl oyster *Pinctada fucata* . Gene. 2014;542: 8–15. 10.1016/j.gene.2014.03.029 24636962

[9] Li X, Bai Z, Luo H, Liu Y, Wang G, Li J. Cloning, differential tissue expression of a novel hcApo gene, and its correlation with total carotenoid content in purple and white inner-shell color pearl mussel *Hyriopsis cumingii* . Gene. 2014;538: 258–265. 10.1016/j.gene.2014.01.046 24486507

[10] Jones DB, Jerry DR, Khatkar MS, Moser G, Raadsma HW, Taylor JJ, et al Quantitative trait loci and genetic association analysis reveals insights into complex pearl quality traits in donor silver-lipped pearl oysters. Aquaculture. 2014;434: 476–485.

[11] Blay C, Sham-Koua M, Vonau V, Tetumu R, Cabral P, Ky CL. Influence of nacre deposition rate on cultured pearl grade and colour in the black-lipped pearl oyster Pinctada margaritifera using farmed donor families. Aquacult Int. 2014;22: 937–953.

[12] Ji L, Liu J, Song W, Li S, Miao D. Effects of dietary Europium complex and Europium(III) on cultured pearl colour in the pearl oyster *Pinctada martensii* . Aquaculture Research. 2013;44: 1300–1306.

[13] Ky C-L, Blay C, Sham-Koua M, Lo C, Cabral P. Indirect improvement of pearl grade and shape in farmed Pinctada margaritifera by donor “oyster” selection for green pearls. Aquaculture. 2014;432: 154–162.

[14] Comfort A. Acid-soluble pigments of shells. 1. The distribution of porphyrin fluorescence in molluscan shells. The Biochemical journal. 1949;44: 111–117. 16748469 PMC1274816

[15] Comfort A. Acid-soluble pigments of molluscan shells. 2. Pigments other than porphyrins. The Biochemical journal. 1949;45: 199–204. 16748612 PMC1274971

[16] Nicholas RE, Comfort A. Acid-soluble pigments of molluscan shells. 4. Identification of shell porphyrins with particular reference to conchoporphyrin. The Biochemical journal. 1949;45: 208–210. 16748614 PMC1274973

[17] Li X, Bai Z, Luo H, Liu Y, Wang G, Li J. Cloning, differential tissue expression of a novel hcApo gene, and its correlation with total carotenoid content in purple and white inner-shell color pearl mussel *Hyriopsis cumingii* . Gene. 2014;538: 258–265. 10.1016/j.gene.2014.01.046 24486507

[18] Kantha SS. Carotenoids of edible molluscs. Journal of Food Biochemistry. 1989;13: 429–442.

[19] Budd A, McDougall C, Green K, Degnan B. Control of shell pigmentation by secretory tubules in the abalone mantle. Frontiers in Zoology. 2014;11: 62.

[20] Bai Z, Zheng H, Lin J, Wang G, Li J. Comparative analysis of the transcriptome in tissues secreting purple and white nacre in the pearl mussel Hyriopsis cumingii. 2013. 10.1371/journal.pone.0053617 PMC3544910 23341956

[21] Wang Z, Gerstein M, Snyder M. RNA-Seq: a revolutionary tool for transcriptomics. Nature reviews Genetics. 2009;10: 57–63. 10.1038/nrg2484 19015660 PMC2949280

[22] Ordas A, Hegedus Z, Henkel CV, Stockhammer OW, Butler D, Jansen HJ, et al Deep sequencing of the innate immune transcriptomic response of zebrafish embryos to Salmonella infection. Fish Shellfish Immunol. 2011;31: 716–724. 10.1016/j.fsi.2010.08.022 20816807

[23] Marie B, Joubert C, Tayale A, Zanella-Cleon I, Belliard C, Piquemal D, et al Different secretory repertoires control the biomineralization processes of prism and nacre deposition of the pearl oyster shell. Proceedings of the National Academy of Sciences of the United States of America. 2012;109: 20986–20991. 10.1073/pnas.1210552109 23213212 PMC3529032

[24] Zhang G, Fang X, Guo X, Li L, Luo R, Xu F, et al The oyster genome reveals stress adaptation and complexity of shell formation. Nature. 2012;490: 49–54. 10.1038/nature11413 22992520

[25] Wang S, Hou R, Bao Z, Du H, He Y, Su H, et al Transcriptome sequencing of Zhikong scallop (Chlamys farreri) and comparative transcriptomic analysis with Yesso scallop (Patinopecten yessoensis). PloS one. 2013;8: e63927 10.1371/journal.pone.0063927 23667690 PMC3646770

[26] Baldo L, Santos ME, Salzburger W. Comparative transcriptomics of Eastern African cichlid fishes shows signs of positive selection and a large contribution of untranslated regions to genetic diversity. Genome biology and evolution. 2011;3: 443–455. 10.1093/gbe/evr047 21617250 PMC3296448

[27] Morton B. Swimming in Amusium pleuronectes (Bivahia: Pectinidae)*. Journal of Zoology. 1980;190: 375–404.

[28] Poutiers JM. Bivalves. (Acephala, Lamellibranchia, Pelecypoda). Carpenter, KE and VH Niem 1998; 123–362.

[29] Grabherr MG, Haas BJ, Yassour M, Levin JZ, Thompson DA, Amit I, et al Full-length transcriptome assembly from RNA-Seq data without a reference genome. Nature biotechnology. 2011;29: 644–652. 10.1038/nbt.1883 21572440 PMC3571712

[30] Blow N. Transcriptomics: The digital generation. Nature. 2009;458: 239–242. 10.1038/458239a 19279641

[31] Tan YD, Xu H. A general method for accurate estimation of false discovery rates in identification of differentially expressed genes. Bioinformatics. 2014;30: 2018–2025. 10.1093/bioinformatics/btu124 24632499

[32] Altschul SF, Madden TL, Schaffer AA, Zhang J, Zhang Z, Miller W, et al Gapped BLAST and PSI-BLAST: a new generation of protein database search programs. Nucleic acids research. 1997;25: 3389–3402. 9254694 10.1093/nar/25.17.3389 PMC146917

[33] Audic S, Claverie JM. The significance of digital gene expression profiles. Genome research. 1997;7: 986–995. 9331369 10.1101/gr.7.10.986

[34] Moore KJ, Swing DA, Copeland NG, Jenkins NA. Interaction of the murine dilute suppressor gene (dsu) with fourteen coat color mutations. Genetics. 1990;125: 421–430. 2379821 10.1093/genetics/125.2.421 PMC1204030

[35] Damek-Poprawa M, Diemer T, Lopes VS, Lillo C, Harper DC, Marks MS, et al Melanoregulin (MREG) modulates lysosome function in pigment epithelial cells. The Journal of biological chemistry. 2009;284: 10877–10889. 10.1074/jbc.M808857200 19240024 PMC2667774

[36] Kim SR, Jang YP, Jockusch S, Fishkin NE, Turro NJ, Sparrow JR. The all-trans-retinal dimer series of lipofuscin pigments in retinal pigment epithelial cells in a recessive Stargardt disease model. Proceedings of the National Academy of Sciences of the United States of America. 2007;104: 19273–19278. 18048333 10.1073/pnas.0708714104 PMC2148280

[37] Andersen SO. Insect cuticular sclerotization: a review. Insect biochemistry and molecular biology. 2010;40: 166–178. 10.1016/j.ibmb.2009.10.007 19932179

[38] Comfort A. The Pigmentation of Molluscan Shells. Biological Reviews. 1951;26: 285–301.

[39] Waite JH. Quinone-tanned scleroproteins. The mollusca. 1983;1: 467–504.

[40] Aguilera F, McDougall C, Degnan BM. Evolution of the tyrosinase gene family in bivalve molluscs: Independent expansion of the mantle gene repertoire. Acta Biomaterialia. 2014. 10.1016/j.actbio.2014.03.031 24704693

[41] Li N, Hu J, Wang S, Cheng J, Hu X, Lu Z, et al Isolation and identification of the main carotenoid pigment from the rare orange muscle of the Yesso scallop. Food chemistry. 2010;118: 616–619.

[42] Elde AC, Pettersen R, Bruheim P, Jarnegren J, Johnsen G. Pigmentation and spectral absorbance signatures in deep-water corals from the Trondheimsfjord, Norway. Mar Drugs. 2012;10: 1400–1411. 10.3390/md10061400 22822381 PMC3397448

[43] Zheng H, Liu H, Zhang T, Wang S, Sun Z, Liu W, et al Total carotenoid differences in scallop tissues of Chlamys nobilis (Bivalve: Pectinidae) with regard to gender and shell colour. Food chemistry. 2010;122: 1164–1167.

[44] Smolenaars MM, Kasperaitis MA, Richardson PE, Rodenburg KW, Van der Horst DJ. Biosynthesis and secretion of insect lipoprotein: involvement of furin in cleavage of the apoB homolog, apolipophorin-II/I. Journal of lipid research. 2005;46: 412–421. 15604521 10.1194/jlr.M400374-JLR200

[45] Mann S. Biomineralization P Concepts in Bioinorganic Materials Chemistry. Oxford: Oxford University Press; 2001.

[46] Green DW, Padula MP, Santos J, Chou J, Milthorpe B, Ben-Nissan B. A Therapeutic Potential for Marine Skeletal Proteins in Bone Regeneration. Marine drugs. 2013;11: 1203–1220. 10.3390/md11041203 23574983 PMC3705399

[47] Weiner S, Addadi L. Crystallization Pathways in Biomineralization. Annual review of materials research. 2011;41: 21–40.

[48] Falini G, Fermani S. Chitin mineralization. Tissue engineering. 2004;10: 1–6. 15009925 10.1089/107632704322791646

[49] Killian CE, Wilt FH. Molecular aspects of biomineralization of the echinoderm endoskeleton. Chemical reviews. 2008;108: 4463–4474. 10.1021/cr0782630 18821807

[50] Blank S, Arnoldi M, Khoshnavaz S, Treccani L, Kuntz M, Mann K, et al The nacre protein perlucin nucleates growth of calcium carbonate crystals. Journal of microscopy. 2003;212: 280–291. 14629554 10.1111/j.1365-2818.2003.01263.x

[51] Klatt AR, Becker AK, Neacsu CD, Paulsson M, Wagener R. The matrilins: modulators of extracellular matrix assembly. The international journal of biochemistry & cell biology. 2011;43: 320–330. 21163365 10.1016/j.biocel.2010.12.010

[52] Marxen JC, Nimtz M, Becker W, Mann K. The major soluble 19.6 kDa protein of the organic shell matrix of the freshwater snail *Biomphalaria glabrata* is an N-glycosylated dermatopontin. Biochimica et biophysica acta. 2003;1650: 92–98. 12922172 10.1016/s1570-9639(03)00203-6

[53] Jiao Y, Wang H, Du X, Zhao X, Wang Q, Huang R, et al Dermatopontin, a shell matrix protein gene from pearl oyster *Pinctada martensii*, participates in nacre formation. Biochemical and biophysical research communications. 2012;425: 679–683. 10.1016/j.bbrc.2012.07.099 22842462

[54] Gao R, Jing P, Ruan S, Zhang Y, Zhao S, Cai Z, et al Removal of off-flavours from radish (Raphanus sativus L.) anthocyanin-rich pigments using chitosan and its mechanism(s). Food chemistry. 2014;146: 423–428. 10.1016/j.foodchem.2013.09.107 24176362

[55] de Vega S, Iwamoto T, Nakamura T, Hozumi K, McKnight DA, Fisher LW, et al TM14 is a new member of the fibulin family (fibulin-7) that interacts with extracellular matrix molecules and is active for cell binding. The Journal of biological chemistry. 2007;282: 30878–30888. 17699513 10.1074/jbc.M705847200

[56] Prasad M, Butler WT, Qin C. Dentin sialophosphoprotein in biomineralization. Connective tissue research. 2010;51: 404–417. 10.3109/03008200903329789 20367116 PMC2933432

[57] Suzuki M, Saruwatari K, Kogure T, Yamamoto Y, Nishimura T, Kato T, et al An acidic matrix protein, Pif, is a key macromolecule for nacre formation. Science. 2009;325: 1388–1390. 10.1126/science.1173793 19679771

[58] Miyamoto H, Miyashita T, Okushima M, Nakano S, Morita T, Matsushiro A. A carbonic anhydrase from the nacreous layer in oyster pearls. Proceedings of the National Academy of Sciences of the United States of America. 1996;93: 9657–9660. 8790386 10.1073/pnas.93.18.9657 PMC38484

[59] Suzuki M, Iwashima A, Tsutsui N, Ohira T, Kogure T, Nagasawa H. Identification and characterisation of a calcium carbonate-binding protein, blue mussel shell protein (BMSP), from the nacreous layer. ChemBioChem. 2011;12: 2478–2487. 10.1002/cbic.201100317 21932217

[60] Isowa Y, Sarashina I, Setiamarga DH, Endo K. A comparative study of the shell matrix protein aspein in pterioid bivalves. J Mol Evol. 2012;75: 11–18. 10.1007/s00239-012-9514-3 22922907

[61] Carafoli E. Intracellular calcium homeostasis. Annual review of biochemistry. 1987;56: 395–433. 3304139 10.1146/annurev.bi.56.070187.002143

[62] Spafford JD, Munno DW, Van Nierop P, Feng ZP, Jarvis SE, Gallin WJ, et al Calcium channel structural determinants of synaptic transmission between identified invertebrate neurons. The Journal of biological chemistry. 2003;278: 4258–4267. 12458203 10.1074/jbc.M211076200

[63] Kimura T, Kubo T. Cloning and functional characterization of squid voltage-dependent Ca2+ channel beta subunits: involvement of N-terminal sequences in differential modulation of the current. Neuroscience research. 2003;46: 105–117. 12725917 10.1016/s0168-0102(03)00038-5

[64] Speiser DI, Loew ER, Johnsen S. Spectral sensitivity of the concave mirror eyes of scallops: potential influences of habitat, self-screening and longitudinal chromatic aberration. J Exp Biol. 2011;214: 422–431. 10.1242/jeb.048108 21228201

[65] Li S, Xie L, Zhang C, Zhang Y, Gu M, Zhang R. Cloning and expression of a pivotal calcium metabolism regulator: calmodulin involved in shell formation from pearl oyster (Pinctada fucata). Comparative biochemistry and physiology Part B, Biochemistry & molecular biology. 2004;138: 235–243. 10.1016/j.cbpc.2004.03.012 15253872

[66] Zayzafoon M, Fulzele K, McDonald JM. Calmodulin and calmodulin-dependent kinase IIalpha regulate osteoblast differentiation by controlling c-fos expression. The Journal of biological chemistry. 2005;280: 7049–7059. 15590632 10.1074/jbc.M412680200

[67] Xie LP, Wu YT, Dai YP, Li Q, Zhang RQ. A novel glycosylphosphatidylinositol-anchored alkaline phosphatase dwells in the hepatic duct of the pearl oyster, *Pinctada fucata* . Mar Biotechnol (NY). 2007;9: 613–623. 17624576 10.1007/s10126-007-9015-3

[68] Weiss IM, Gohring W, Fritz M, Mann K. Perlustrin, a Haliotis laevigata (abalone) nacre protein, is homologous to the insulin-like growth factor binding protein N-terminal module of vertebrates. Biochemical and biophysical research communications. 2001;285: 244–249. 11444832 10.1006/bbrc.2001.5170

[69] Setiamarga DH, Shimizu K, Kuroda J, Inamura K, Sato K, Isowa Y, et al An in-silico genomic survey to annotate genes coding for early development-relevant signaling molecules in the pearl oyster, Pinctada fucata. Zoolog Sci. 2013;30: 877–888. 10.2108/zsj.30.877 24125651

[70] Mouries LP, Almeida MJ, Milet C, Berland S, Lopez E. Bioactivity of nacre water-soluble organic matrix from the bivalve mollusk *Pinctada maxima* in three mammalian cell types: fibroblasts, bone marrow stromal cells and osteoblasts. Comparative biochemistry and physiology Part B, Biochemistry & molecular biology. 2002;132: 217–229. 10.1016/s1096-4959(01)00524-3 11997223

[71] Huang XD, Liu WG, Guan YY, Shi Y, Wang Q, Zhao M, et al Molecular cloning, characterization and expression analysis of tumor necrosis factor receptor-associated factor 3 (TRAF3) from pearl oyster *Pinctada fucata* . Fish Shellfish Immunol. 2012;33: 652–658. 10.1016/j.fsi.2012.06.026 22796485

[72] Miyashita T, Hanashita T, Toriyama M, Takagi R, Akashika T, Higashikubo N. Gene cloning and biochemical characterization of the BMP-2 of *Pinctada fucata* . Bioscience, biotechnology, and biochemistry. 2008;72: 37–47. 18175919 10.1271/bbb.70302

[73] Yan F, Luo S, Jiao Y, Deng Y, Du X, Huang R, et al Molecular characterization of the BMP7 gene and its potential role in shell formation in *Pinctada martensii* . International journal of molecular sciences. 2014;15: 21215–21228. 10.3390/ijms151121215 25407527 PMC4264221

[74] Thiel T, Michalek W, Varshney RK. Exploiting EST databases for the development and characterization of gene-derived SSR-markers in barley (Hordeum vulgare L.). Theoretical and Applied Genetics. 2003;106: 411–422. 12589540 10.1007/s00122-002-1031-0

