# Supplementary material for: Mantle Branch-Specific RNA Sequences of Moon Scallop Amusium pleuronectes to Identify Shell Color-Associated Genes
Source: PLoS One. 2015 Oct 23;10(10):e0141390. doi: 10.1371/journal.pone.0141390 (PMC4619886; doi:10.1371/journal.pone.0141390)
Supplement: S1 Table — (DOCX) [file pone.0141390.s005.docx]

**Tables 1. Primer sequences for RT-PCR**

| **geneID** | **Sequence** |
| --- | --- |
| Unigene122474_All(S) | CAGATGGTGTTACCGACTTGGG |
| Unigene122474_All(A) | CGTGTTCTTTGATTTGCGTTTT |
| Unigene1888_All(S) | CACAGCCTCCACCAACACTATT |
| Unigene1888_All(A) | CTCCCCCGCCCCCAT |
| Unigene66754_All(S) | TAGCAGCGTTTCCTCCAGATACC |
| Unigene66754_All(A) | TTTGCCGATTTGGTTCTCTATTT |
| Unigene18915_All(S) | ATGAGGTTTGTGCTGGAGAGG |
| Unigene18915_All(A) | TTGTGAACTATGAAGTGAGTGAATG |
| Unigene55171_All(S) | TGGGAAAGTCATAGCAAC |
| Unigene55171_All(A) | CCCTGCGTTGTTAGTTAG |
| Unigene17708_All(S) | GCACTTGCGGAACACCTCA |
| Unigene17708_All(A) | GGCGGGGGAACAATGGAC |
| Unigene51590_All(S) | GTGTTTGGTTGCGTGTTGTGT |
| Unigene51590_All(A) | CGATGCGATGTCCTTGCTG |
| Unigene77517_All(A) | TCCCGATGAAGTCCAAAACA |
| Unigene77517_All(S) | TACAACTGCTGGCACCACCT |
| Unigene65582_All(A) | CACCGCCCCCTCAAACTAA |
| Unigene65582_All(S) | CGTCACACTTCCTCGCCTTCT |
| Unigene45469_All(S) | GTGTTGCCTGTAGCGTTGGGT |
| Unigene45469_All(A) | GATGTGCGTCCGTATTCTGGT |
| Unigene81413_All*(S) | CAGAAAGGAGGTGCCAAGAAG |
| Unigene81413_All*(A) | GCAAGAGGAGCCAAGCAGTTA |

* represents the reference gene GAPDH.
