# Supplementary material for: Mantle Branch-Specific RNA Sequences of Moon Scallop Amusium pleuronectes to Identify Shell Color-Associated Genes
Source: PLoS One. 2015 Oct 23;10(10):e0141390. doi: 10.1371/journal.pone.0141390 (PMC4619886; doi:10.1371/journal.pone.0141390)
Supplement: S2 Table — (DOCX) [file pone.0141390.s006.docx]

**Table S2. Sequencing and assembling of the unigenes identified in this study.**

A. Output statistics of sequencing

| Samples | Clean Reads | Total Nucleotides  (nt) | Q20 percentage | N percentage | GC percentage |
| --- | --- | --- | --- | --- | --- |
| RS | 54,361,178 | 4,892,506,020 | 96.28% | 0.01% | 40.58% |
| WS | 50,796,780 | 4,571,710,200 | 96.35% | 0.01% | 42.19% |

B. The length distribution of the Unigene(nt)

| sample |  | 100-500 | 500-1000 | 1000-1500 | 1500-2000 | >=2000 | N50 | Mean | All Unigene |
| --- | --- | --- | --- | --- | --- | --- | --- | --- | --- |
| RS-unigene | number | 127,264 | 30,502 | 9,490 | 4,279 | 5,117 | 698 | 514 | 176,652 |
|  | percent | 72.04% | 17.27% | 5.37% | 2.42% | 2.90% |  |  |  |
| WS-unigene | number | 106,084 | 25,458 | 8,505 | 4,137 | 5,191 | 764 | 536 | 149,375 |
|  | percent | 71.02% | 17.04% | 5.69% | 2.77% | 3.48% |  |  |  |
| All -unigene | number | 99,458 | 33,473 | 11,781 | 6,015 | 8,794 | 979 | 669 | 159,521 |
|  | percent | 62.35% | 20.98% | 7.39% | 3.77% | 5.51% |  |  |  |

C. Gap distribution (N/length) %

| sample | number/percent | 0%~5% | 5%~20% | 20%~40% | 40%~60% | 60%~80% | 80%~100% |
| --- | --- | --- | --- | --- | --- | --- | --- |
| RS | number | 232,480 | 0 | 0 | 0 | 0 | 0 |
|  | percent | 100.00% | 0.00% | 0.00% | 0.00% | 0.00% | 0.00% |
| WS | number | 295,049 | 0 | 0 | 0 | 0 | 0 |
|  | percent | 100.00% | 0.00% | 0.00% | 0.00% | 0.00% | 0.00% |
